# Challenges and recommendations to improve implementation of phototherapy among neonates in Malawian hospitals

**DOI:** 10.1186/s12887-022-03430-y

**Published:** 2022-06-27

**Authors:** Mai-Lei Woo Kinshella, Sangwani Salimu, Brandina Chiwaya, Felix Chikoti, Lusungu Chirambo, Ephrida Mwaungulu, Mwai Banda, Tamanda Hiwa, Marianne Vidler, Elizabeth M. Molyneux, Queen Dube, Joseph Mfutso-Bengo, David M. Goldfarb, Kondwani Kawaza, Alinane Linda Nyondo-Mipando

**Affiliations:** 1 Department of Obstetrics and Gynaecology, BC Children’s and Women’s Hospital and University of British Columbia, Vancouver, Canada; 2 Department of Health Systems and Policy, School of Global and Public Health, Kamuzu University of Health Sciences, Blantyre, Malawi; 3 Department of Pediatrics and Child Health, Kamuzu University of Health Sciences, Blantyre, Malawi; 4 Queen Elizabeth Central Hospital, Pediatrics, Blantyre, Malawi; 5 Center of Bioethics for Eastern and Southern Africa (CEBESA), Blantyre, Malawi; 6 Department of Pathology and Laboratory Medicine, BC Children’s and Women’s Hospital and University of British Columbia, Vancouver, Canada

**Keywords:** Phototherapy, Neonatal jaundice, Neonates, Preterm births

## Abstract

**Background:**

Severe neonatal jaundice can result in long term morbidities and mortality when left untreated. Phototherapy is the main-stay intervention for treating moderate jaundice and for prevention of the development of severe jaundice. However, in resource-limited health care settings, phototherapy has been inconsistently used. The objective of this study is to evaluate barriers and facilitators for phototherapy to treat neonatal jaundice at Malawian hospitals.

**Methods:**

We conducted a convergent mixed-method study comprised of a facility assessment and qualitative interviews with healthcare workers and caregivers in southern Malawi. The facility assessment was conducted at three secondary-level hospitals in rural districts. In-depth interviews following a semi-structured topic guide were conducted at a district hospital and a tertiary-level hospital. Interviews were thematically analysed in NVivo 12 software (QSR International, Melbourne, Australia).

**Results:**

The facility assessment found critical gaps in initiating and monitoring phototherapy in all facilities. Based on a total of 31 interviews, participants identified key challenges in diagnosing neonatal jaundice, counselling caregivers, and availability of infrastructure. Participants emphasized the need for transcutaneous bilirubinometers to guide treatment decisions. Caregivers were sometimes fearful of potential harmful effects of phototherapy, which required adequate explanation to mothers and family members in non-medical language. Task shifting and engaging peer support for caregivers with concerns about phototherapy was recommended.

**Conclusion:**

Implementation of a therapeutic intervention is limited if accurate diagnostic tests are unavailable. The scale up of therapeutic interventions, such as phototherapy for neonatal jaundice, requires careful holistic attention to infrastructural needs, supportive services such as laboratory integration as well as trained human resources.

**Supplementary Information:**

The online version contains supplementary material available at 10.1186/s12887-022-03430-y.

## Background

Approximately 60% of infants born at term and 80% of premature infants develop jaundice in the first week of life [1]. Neonatal jaundice results from a build-up of bilirubin in the blood, culminating in a yellowish discolouration of the sclera and the skin [1–4]. Bilirubin is mainly a product of the breakdown of old or damaged haemoglobin in red blood cells that are cleared from tissues by the liver. Neonatal jaundice in the absence of blood group incompatibility, haemolysis, sepsis, birth trauma and metabolic disorders usually resolves without serious complications within a week [1–4]. High levels of unconjugated bilirubin, exacerbated by the inability of a premature baby’s liver to clear the bilirubin, can result in long term morbidities and [1–5]. These complications include kernicterus, also known as acute and chronic bilirubin encephalopathy, which involves the accumulation of unconjugated bilirubin in the brain causing irreversible brain damage and consequent developmental delay, cerebral palsy, hearing loss, visual and dental problems [1–5].

While the number of severe neonatal jaundice cases in high-income countries (HICs) has decreased significantly since the 1990s with improvements in prevention, the rate in low- and middle-income countries (LMICs) has not [5]. LMICs, particularly in sub-Saharan Africa and South Asia, carry a disproportionate burden in both incidence of and deaths associated with severe neonatal jaundice [6, 7]. The incidence of severe neonatal jaundice is 244.1 per 10,000 live births in LMICs compared to 3.7 per 10,000 live births in HICs, with the Africa region having highest incidence at 667.8 per 10,000 live births [6]. Of the 114,000 neonatal deaths associated with severe neonatal jaundice in 2010, 35% were in sub-Saharan Africa and 39% in South Asia [7].

Serious adverse effects of neonatal jaundice are largely preventable if severe jaundice is identified early and treated promptly with effective phototherapy and/or exchange blood transfusion for very severe cases [1–5, 8, 9]. Along with the management of Rhesus incompatibility, phototherapy has helped to greatly reduce the need for exchange blood transfusions in HICs, which require highly skilled personnel and are associated with the risk of complications that accompany a blood transfusion [8, 9]. High rates of avoidable exchange blood transfusions are reported in LMICs. For example, a Nigerian study found approximately one in 20 infants admitted into the neonatal intensive care unit underwent exchange blood transfusions, 51.8% of which could have been avoided with intensive phototherapy [8].

Phototherapy was recognized as a standard of care for neonatal jaundice in the 1980s and guidelines for use were circulated by the American Academy of Pediatrics in 1994, however, the use of phototherapy has been inconsistent in LMICs [10, 11]. Consequently, there is a need to understand issues around implementation and scaling up of the technology in resource limited health settings, especially in sub-Saharan Africa. In Malawi, phototherapy units are available at tertiary hospitals located in urban centres and recently have been provided to secondary level district hospitals [12, 13]. However, the absence of reliable and timely serum bilirubin results was reported to challenge phototherapy utilization at a tertiary hospital in southern Malawi [12]. There is a current gap in the literature on implementation factors for phototherapy at the secondary-level district hospitals. The purpose of this study was to evaluate barriers and facilitators for phototherapy to treat neonatal jaundice at tertiary- and secondary-level hospitals in Malawi.

## Method

### Study design and research setting

We conducted a convergent mixed-method study, which employed both quantitative and qualitative data collection approaches separately and together at the point of interpretation to highlight the areas where the two data sets converge or diverge [14]. This study drew from a cross-sectional quantitative facility assessment on the availability and quality of neonatal care at district level hospitals in southern Malawi [13] and qualitative interviews with health workers and caregivers on their perspectives and experiences of phototherapy. This study was part of the larger project, “Integrating a neonatal healthcare package for Malawi”, funded as part of the Innovating for Maternal and Child Health in Africa (IMCHA) initiative by the Canadian International Development Research Centre, Global Affairs Canada and the Canadian Institutes for Health Research.

Study sites consisted of a secondary-level hospital and a tertiary hospital in southern Malawi. Both hospitals are public government hospitals. The secondary-level hospital represented the highest level of clinical care available for the rural district, while the tertiary hospital was a large regional referral facility located in an urban centre. All hospitals in the study provided inpatient neonatal care services and had separate areas for admitted neonates.

### Data collection

The facility assessment employed the WHO Integrated Maternal, Neonatal, and Child Quality of Care Assessment and Improvement Tool adapted for use on neonates. Researchers first met with hospital management and clinical staff to introduce the project, engaged in a walk-through of the facility, assessed service availability and quality following a checklist guide and observations, then met with hospital representatives to discuss preliminary findings and action points. The assessment was completed in November 2017 and more information on the methodology is described in our earlier paper [13].

Participants for the qualitative interviews were purposively sampled to include healthcare workers (HCWs) engaged in service delivery or supervision of clinical newborn care and caregivers of neonates undergoing phototherapy. HCWs included nurses, clinicians, pediatric physicians and district health managers (district health officer, district medical officer, district nursing officer). Caregivers included mothers, fathers and relatives accompanying mothers during their infant’s admission at the hospital. Interviews were conducted either in-person or over the phone by certified nurse-midwife-technicians (authors BC, FC, and EM) and two public health specialists (authors SS and LC). Interviews followed a semi-structured topic guide (Additional file 1), were 30 to 60 min in length and were conducted in either the local language of Chichewa or English as preferred by each participant. There were no refusals to participate nor repeat interviews. Interviews were conducted between February and August 2020, with an interim review conducted between March and June 2020 to assess saturation and establish protocols to prevent transmission of COVID-19. Due to the COVID-19 pandemic and upon reviewing transcripts for comprehensiveness of content, the study team decided that data saturation was achieved with a tertiary hospital and a district hospital. Three further interviews were conducted via phone with HCWs to complete these sites.

### Data analysis

Facility assessment data were first collected on paper, then scanned and transferred to a REDCap database (Vanderbilt University, Nashville, USA) by two independent researchers double entering the data for data quality control. Following the WHO Integrated Maternal, Neonatal, and Child Quality of Care Assessment and Improvement Tool guidance, aspects of care were scored on a Likert scale from one to five [13, 15, 16]. A score of one indicated that services were not provided or were dangerously inadequate, a score of two indicated a considerable need for improvement, a score of three indicated some need for improvement, while scores of four or five were considered acceptable with little improvements needed or meet standards of care, respectively.

Interviews were digitally recorded with participants’ permission and transcribed in verbatim. Interviews conducted in Chichewa were translated into English. Transcripts were uploaded to NVivo12 (QSR International, Melbourne, Australia). We analyzed the data thematically following a six-step process of first familiarizing with the data, coding transcripts, generating themes from coded data, reviewing then naming themes and lastly writing up [17]. MWK and ALNM developed a coding guide following a discussion of the pilot transcripts and all data were coded by SS under the supervision of MWK and ALNM. Themes were discussed and refined by SS, MWK and ALNM and verified against the audios to ensure they correctly reflected the data.

### Ethical considerations

Ethical approvals were obtained from the research ethics boards of the Malawi College of Medicine (P.08/15/1783) and the University of British Columbia (H15–01,463-A003) and all methods were performed in accordance with the relevant guidelines and regulations. Informed consent was obtained prior to interviews. Hospitals and participants were assigned a unique identifier and participant groups were generalized (i.e. district health management officials rather than district health officer) to help maintain confidentiality.

## Results

The facility assessment and interviews converged to highlight challenges in implementing phototherapy within Malawian hospitals. There was little divergence between the qualitative and quantitative data, rather that HCW and caregiver perspectives illuminated the context of gaps found in the facility assessment and provided potential solutions.

### Facility assessment

The facility assessment found critical gaps in identifying and treating neonatal jaundice at the district hospital (Table 1). There was a considerable need for improvement in examining neonates for jaundice and phototherapy guidelines were not used by HCWs. Severe jaundice was not always recognized or appropriately managed, and documentation was poor requiring considerable need for improvement. While bilirubin testing was reported to be available at the laboratory, there were no procedures to check bilirubin level. One LED phototherapy lamp was available, but did not have regular maintenance to check for correct functioning.

**Table 1:** Facility assessment of the district hospital around neonatal jaundice and phototherapy

	**District hospital**
**Examination and management procedures**	
All babies are examined for jaundice	2
Procedures are in place to check the bilirubin level	1
Severe jaundice is recognized and appropriately managed	3
Phototherapy and guidelines when to use it are available and adequate hydration is ensured	1
Documentation on absence or presence of jaundice kept	2
**Laboratory**	
Is bilirubin testing available?	Yes
If available, indicate average time to get bilirubin results	About 45 min—1 h
**Equipment and maintenance**	
Is there a phototherapy lamp available?	Yes (1 lamp available)
Phototherapy lamps are checked regularly for correct functioning	1

1- Services not provided or were dangerously inadequate; 2—Considerable need for improvement; 3—Some need for improvement; 4—Little improvements needed; 5—Meets standards of care

### Qualitative findings

#### Participant characteristics

There were 31 interviews conducted including 19 at the tertiary hospital and 12 from a district hospital. From the tertiary hospital, 10 HCWs interviewed included nursing officers, clinical officers, physicians and nurses. There were 9 caregivers interviewed at the tertiary hospital including grandmothers, mothers and fathers. From the district hospital, 9 HCWs interviewed including nursing officers, clinical officers, district health management officials and nurses. Three caregivers interviewed at the district hospital included mothers and a father.

**Fig. 1:**
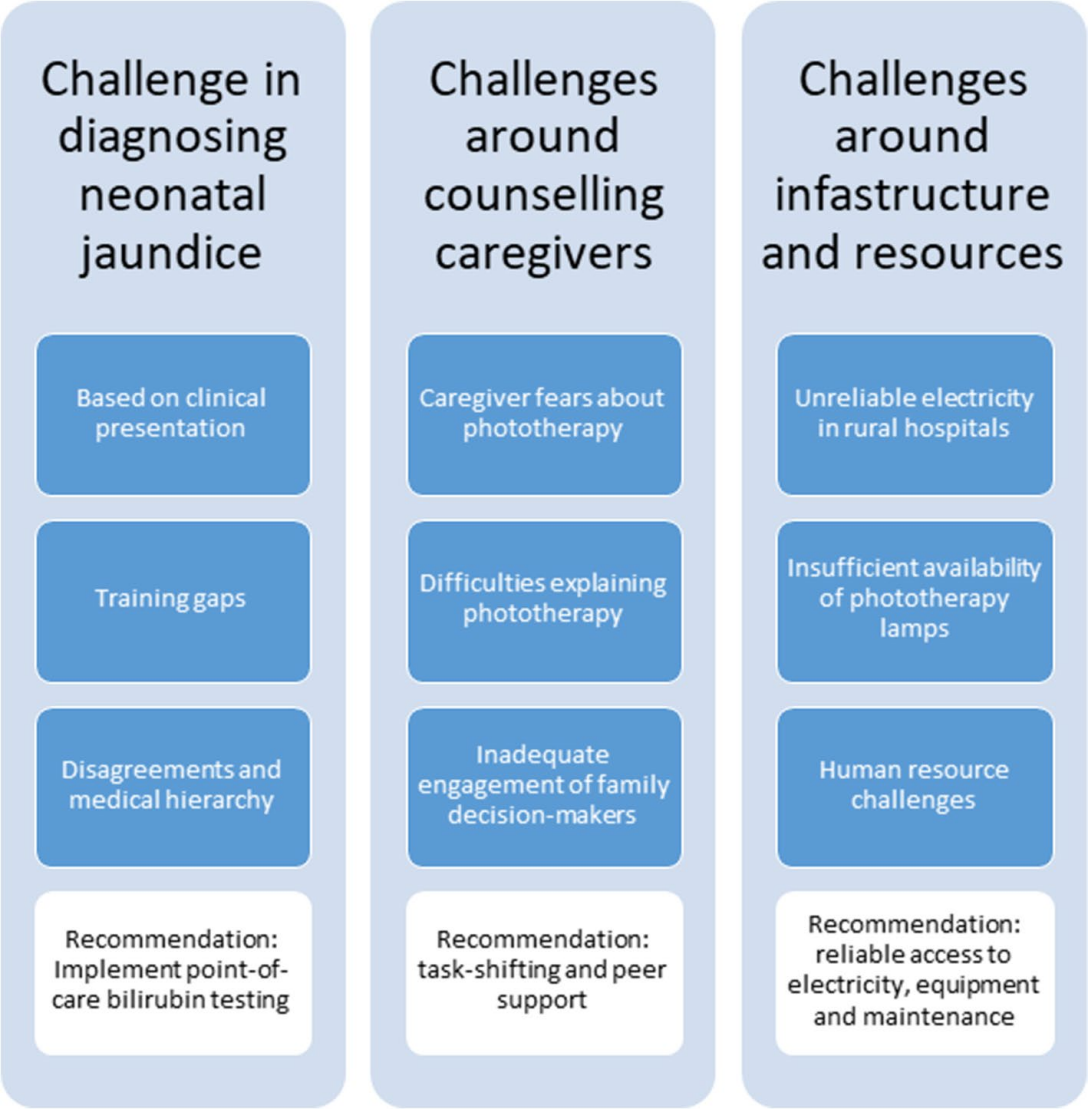
Challenges and recommendations for phototherapy implementation for neonates in Malawian hospitals

Both the tertiary and district hospital used LED phototherapy. There were three main areas of challenges around the effective implementation of phototherapy for the management of neonatal jaundice shared by participants illustrated in Fig. 1 (see also Additional file 2). These included the challenge of diagnosing neonatal jaundice, inadequate counselling to alleviate caregiver fears about phototherapy, and infrastructure and resource gaps.

#### The challenge of diagnosing neonatal jaundice

##### Diagnosing clinical presentation

Healthcare workers at both tertiary and district hospitals reported that diagnosing neonatal jaundice was a critical challenge. Transcutaneous bilirubinometers (TcBs), a non‐invasive point-of-care device that estimates serum bilirubin level by directing light into the skin and measuring the intensity of the returning wavelength, were not available at either site. Healthcare workers described that they instead diagnosed neonatal jaundice on clinical presentation, such as by checking the infant’s skin colour by pressing the forehead or chest, or by urine discolouration. Objective diagnostic tools were reported to be unavailable even at the tertiary hospital.

“It’s a bit tricky because we don’t have, transcutaneous bilirubinometer that helps us to identify whether the child needs phototherapy or not. Uhh currently, we are using our eyes…” Tertiary hospital nurse

##### Training gaps

While guidelines on phototherapy indicators were available and posted on walls, healthcare workers shared that training gaps resulted in poor understanding of guidelines. Only a few staff received formal training, with the expectation that they would train others on their teams. However, on-the-job training was described as brief and lacking comprehensiveness. Training gaps contributed to delays in initiating phototherapy.

“Not really knowing what jaundice look like … because we were not trained. So everyone rates [the] baby on their own, so the challenge is that to some, it is very difficult to identify the jaundice… Of [the] nurses in our ward, it’s only one person who have trained on phototherapy and the other four we did not go to training, so it’s a big challenge” District hospital nurse

Informal, on-the-job training that oriented healthcare workers to the use of the device without comprehensive knowledge on neonatal jaundice and the use of phototherapy contributed to gaps in understanding guidelines. Also, sometimes guidelines referred to bilirubin levels, which made them irrelevant for practical use on the ward where capacity to test is limited.

“I have seen there is a coding guideline in the nursery where they have documented the values if you are using whether a bilibrinometer or bilirubin levels from the lab and also… where you can check. [For example,] maybe if you can see that this part is looking more jaundiced… If you ask most of the people… they don’t really understand most of it… I don’t think they (the guidelines) are really used.” Tertiary hospital physician

##### Disagreements and medical hierarchy

Healthcare workers at both tertiary and district hospitals also shared that there was sometime disagreement on the clinical indicators between nurses and physicians/clinical officers. Nurses shared that because they were more involved in routine monitoring and follow-up, they were more sensitive to slight changes in skin colour though clinicians had more clinical decision-making power. Nurses called for improved bilirubin diagnostics to help resolve these conflicts in diagnosing neonatal jaundice and inform appropriate phototherapy use.

“Maybe a nurse has seen a child maybe while giving medicine that this child is looking like jaundice [and recommends] we should start him on phototherapy, then you find the doctor has seen him and says no he is fine, he should be let out…. The doctor has more power than the nurse, but most of the time, it is that the nurse is working more closely with the patients than the doctors. The doctors can see the child today then he will see him again when, maybe tomorrow his friend will see him, but as a nurse she will see him today, see him tomorrow so you can differentiate that this child, at first he looked pink, here we see that he has changed…. But if … they measure the bilirubin… so the results are what determine how we manage the child” Tertiary hospital nurse

#### Challenges around counselling caregivers on phototherapy

##### Caregiver fears about phototherapy

Reported by participants from both the urban tertiary hospital and the rural district hospital, there were fears among caregivers that phototherapy was dangerous for their infants.

“They were just concerned that isn’t this dangerous? Since people, when they hear that a child has gone to phototherapy, they are afraid, so it just scared them” Mother from the tertiary hospital

There were fears around harmful effects of phototherapy, including that that the blue light was destructive to the infant, that it could destroy the infant’s reproductive organs in particular, or that it sucked the blood out of the infant.

“Most people … think if we put a baby on that machine it will die. For example, if we tell them of the oxygen machine, they say the baby will die. The same thing with this machine (phototherapy); they think the baby will die.” District hospital nurse

“I was asking other women, I said, when you are looking at this bulb, how do you look at it? They said, it sucks blood…They say it sucks blood, and the child does not get well.” District hospital nurse

“Others say that if a child is on these lights he will never give birth, his what is destroyed, his reproductive organs.” Tertiary hospital nurse

##### Difficulty in explaining phototherapy to caregivers

Healthcare workers shared that it was difficult to explain phototherapy in non-medical language that caregivers could understand. The vocabulary around phototherapy made it difficult to explain, which is compounded by training gaps among staff who may not fully understand how phototherapy functions themselves.

“I think the vocabulary. They don’t really understand how this light works and that becomes difficult. ” District health management official

##### Inadequate engagement of family decision-makers

Another challenge was that healthcare workers mainly counselled mothers, but other family members such as mothers-in-law and husbands are influential in decision-making. A father reflected that he felt marginalized from the care of his infant because men have very limited access to the neonatal ward.

“I wouldn’t know because as husbands here, we are only given little time just 5 minutes to go and see the baby… it is very limited. I as a father, the experience that I got here is that, as men we are not permitted to have more time there.” Father from the tertiary hospital

“Most of the time, we explain to the mothers…[but] they may ask from other guardians’ relatives like their mothers or their in-laws.” District hospital nurse

#### Infrastructure and resource gaps

##### Unreliable electricity at rural hospitals

A major challenge at the rural district hospitals was unreliable electricity. Electrical outages caused delays in initiation phototherapy for infants.

“Sometimes electricity…Because here in our unit, when power goes off, it’s a problem. We don’t have a generator.” District hospital clinical officer

##### Insufficient availability of phototherapy lamps

While the urban tertiary hospital had reliable electricity, overcrowding challenged the availability of phototherapy lamps. Insufficient availability led to doubling up a single phototherapy lamp with two infants. There may also be delays in initiation or premature removal of the infant from phototherapy if another infant presents with more severe jaundice.

“There [was] also another child. They left him (her child) and helped the other one first…This delay was maybe because the other child’s condition was worse than mine.” Mother from the tertiary hospital

##### Human resource shortages

Nursing and clinician shortages were major challenges in both tertiary and district hospitals. A high patient to nurse ratio was associated with irregular monitoring of infants on phototherapy.

“I think that they (staff) are trained (to monitor) but …I think that they are too short staffed, what I mean is that there are too many babies for the staff. So the work sometimes becomes overwhelming and sometime you need assistance, some would assist you but some will be angry when you are talking to them, so you just leave them to do their work, but sometimes you need more of their assistance…” Father at the tertiary hospital.

There was no clinician dedicated to the neonatal ward in the district hospital. Even with nurses dedicated to the neonatal ward in the district hospital, overall nursing shortages at the hospital means that they may be pulled into other wards when they are busy.

“Not having enough human resource. We don’t have a clinician based in nursery full time. We don’t have enough nurses, of course there are there that are allocated in the nursery but they have to look for all the babies in the nursery. Sometime they might delay monitoring the baby because the thing they are doing is an emergency or it’s a priority.” District health management official

#### Recommendations to improve implementation of phototherapy

Regarding diagnosis, participants recommended the use of bilirubin testing as an objective measure of neonatal jaundice.

“… just looking at the baby clinically is quite subjective, I think that why sometimes we do have maybe some disagreements, say no this one is very jaundiced somebody says is not, so I think if we can have some bilirubin measuring machines that would be very important “ Tertiary hospital clinical officer

Regarding counselling caregivers, participants recommended task-shifting and engaging peer support for caregivers with concerns about phototherapy. Healthcare workers shared that hesitant mothers who spoke with another mother who had an infant on phototherapy often helped to alleviate their fears.

“If you have a guardian who her baby was once on phototherapy, we involve them to explain to that guardian who is not willing to help them understand how important it is.” District hospital nursing officer

Reliable electricity was a barrier to phototherapy as well as other neonatal interventions at the district hospitals. Though overcrowding was more of a concern at the tertiary hospital, healthcare workers at the district hospital shared that the availability of only one phototherapy lamp limited their ability to treat neonatal jaundice and more lamps would help facilitate service delivery. Reliable access to pads to cover infant’s eyes during phototherapy was also recommended, as they are currently improvising with cotton and gauze, which was unreliable.

“Mostly… the issue of power is a problem. The availability of a generator would be helpful, so that this process should be continuous. As a nursery, we will not benefit through phototherapy alone, CPAP will also benefit” District hospital clinical officer

“We don’t have enough phototherapy light, that’s a challenge for us and material to cover the eyes…” District health management official

Participants shared that the phototherapy lamps were often functional and having maintenance teams available at the hospitals if any of the machines have a problem was identified as a facilitator to implementation.

“We called maintenance team to come and fix it the same day… Most of the guys live within the hospital. Even at night, we call them.” District hospital nurse

## Discussion

Taken together, the facility assessment and interviews revealed that challenges in identifying neonatal jaundice in the absence of TcB devices was a critical barrier to the appropriate initiation of phototherapy. While hospital laboratory bilirubin testing and phototherapy guidelines were often reported available, there were gaps in understanding and using them to check bilirubin levels and guide therapy. Additionally, difficulties in counselling caregivers who may be fearful of potential harms, poor engagement of family decision-makers, and inadequate equipment and supplies were identified as initiation barriers. Staffing shortages were a critical barrier to regular monitoring of infants undergoing phototherapy. Strengthening capacities to test bilirubin, engaging peers to support counselling caregivers, reliable electricity supplies, increased availability of phototherapy lamps and eye covering pads and on-site maintenance teams were recommended to improve phototherapy implementation.

Our study findings are in line with previous literature that reported delays in initiating phototherapy at health facilities providing specialized neonatal care as a major challenge in effective phototherapy use in resource-constrained settings [10]. Barriers to effective phototherapy use reported by previous research include a lack of guidelines for the management of neonatal jaundice and bilirubin measurement tools, an erratic power supply, frequent equipment breakdowns, inadequate skin exposure from overcrowding with multiple infants placed under a single device and sub-optimal irradiance levels [8, 18–20]. Our study found that while guidelines were often available, they were underutilized due to knowledge gaps among HCWs or guidelines that referred to bilirubin levels were not relevant due to lack of testing.

The lack of an objective measure to diagnose neonatal jaundice emerged as a fundamental barrier in the effective implementation of phototherapy in our study. There is some controversy as to the reliability of use of visual inspection (e.g. through use of Kramer’s rule) in the assessment of neonatal jaundice, however several studies conducted with children with darker pigmented skin have demonstrated relatively low sensitivity relative to laboratory diagnosis [21–23]. The importance of integration between the laboratory and clinical care wards is also highlighted in our study. While the facility assessment confirmed that the district hospital laboratory had capacity to perform serum bilirubin tests, HCWs interviewed reported a lack of bilirubin testing even at the tertiary hospital. More research is needed to explore this disconnect in more depth, which compromises ability to identify neonatal jaundice and support effective initiation of phototherapy, as well as to support the use of point-of-care TcB devices.

Previous research at a large tertiary hospital in southern Malawi between 2010–2011 found that TcB readings can effectively guide phototherapy treatment for neonates in resource-limited health settings, especially guiding decisions to initiate phototherapy [12]. However, our research a decade later at the same facility found that TcB devices have yet to be routinely implemented. A reason may be because TcBs are very expensive, about $5,000 USD each, and need to be looked after carefully, which reduces their feasibility in a low-resource health setting. Other innovative point of care devices that may be more feasible include the BiliDx, which is a handheld device that can measure total bilirubin from a heel prick blood sample, requires minimal training and skill in its use and was evaluated to be more accurate than a TcB in Malawi [24], as well as smartphone apps like Bilicam [25]. These devices are more affordable and may be more feasible, particularly in the district hospital where phototherapy is used less frequently.

Phototherapy initiation challenges were discussed more in our study than the quality of intervention delivery, or device maintenance. For example, while overcrowding was discussed by participants in our study, it was reported in reference to insufficient lamps to initiate all infants requiring phototherapy rather than inadequate skin exposure. Sub-optimal irradiance was not discussed and HCWs reported that phototherapy lamps were largely functional at their facilities. Though phototherapy lamps were not regularly checked for function, maintenance teams were available if problems arose. The use of LED bulbs for phototherapy may also contribute to lower maintenance needs reported in our study. Current evidence suggests that LED bulbs in devices are as effective as other light sources and have special advantages in LMICs as they are more affordable, more power efficient, portable, weigh less, have a longer life span and produce less heat. The bulbs have been shown to be effective for at least one year of continuous use [26–28]. Older phototherapy devices, often those donated from high-resource settings, frequently broke down due capacity gaps to perform regular product maintenance and required special expensive bulbs [28].

Our study also emphasized the challenges around human resource shortages and the need to effectively engage caregivers, which has also been reported in the scale up of other neonatal interventions in LMICs. Descriptions of inadequate monitoring within staffing shortages as staff focus on emergency cases has been reported with kangaroo mother care (KMC), breastfeeding promotion, and bubble continuous airway pressure (CPAP) [29–31]. However, unlike with KMC or breastfeeding, irregular follow-up may be especially worrisome to caregivers for phototherapy because of fears that the blue light is actively harming the infant. Concerns that infants put on phototherapy would die were similar to fears around other neonatal technologies such as bubble CPAP [32]. A study on caregiver perceptions of bubble CPAP in Malawi found that caregivers benefit from regular reassurance from medical staff and by explaining how the technology worked with simply props to promote better understanding [32]. While a pictorial guide for HCWs has been previously descried to support phototherapy implementation [10], simple illustrations with a script for counselling caregivers in non-medical language may also be helpful, especially as HCWs report difficulties in translating technical vocabulary into explanations that can be understood by caregivers. Also, implementation of a pictorial guide with a script to explain phototherapy can increase the quality of counselling by standardizing content and support task-shifting to peer support workers. Implementation of a pictorial guide and task-shifting may help reduce both human resource and caregiver engagement challenges, and more research is needed to explore these.

A strength of this study is the triangulation of findings from the facility assessment and qualitative interviews. While only one district hospital from the facility assessment was included in the current study to match the qualitative component, comparisons with two other district-level hospitals, including a government and a private not-for-profit mission hospital, found similar gaps in phototherapy care suggesting a degree of generalizability, at least within the Malawian context. However, the study is limited by conducting the facility assessment only at district hospitals and not at the tertiary hospital. Additionally, because phototherapy was used less frequently at the district hospital in comparison to the tertiary hospital, there were fewer caregivers available with experience of phototherapy being initiated with their infants. This restricted our sample of caregivers at the district hospital. Also, there was potential sampling bias of caregivers. Because only caregivers with infants currently admitted at the hospital with experience of phototherapy were interviewed, perspectives of caregivers who refused is missing. Consequently, our understanding of parental fears around phototherapy is largely indirect as shared by healthcare workers. Furthermore, data collection was stopped early due to the COVID-19 pandemic. While our research team assessed the transcripts for saturation, there is a possibility that some issues may have been missed. Lastly, our study is limited to LED phototherapy. Further research can explore the effectiveness and feasibility of other innovative forms of phototherapy in resource-limited settings, such as filtered sunlight and solar powered phototherapy.

## Conclusion

A key lesson learned in the current study on the phototherapy in resource-limited Malawian hospitals, with implications for the scale up of neonatal innovations more broadly, is that the use of a therapeutic intervention is limited if accurate diagnostic processes are not implemented in parallel. Participants recommended the use of point-of-care bilirubin testing devices to provide objective indicators of neonatal jaundice for the effective application of phototherapy, alongside overall strengthening in terms of staffing, training and reliable access to essential equipment and supplies. The scale up of therapeutic interventions, such as phototherapy, require careful holistic attention to infrastructural needs, supportive services such as laboratory integration, and supportive staffing policies to ensure adequately trained staff and less rotation between wards to improve the quality of care.

## Supplementary Information

**Additional file 1. **Caregiver experiences with phototherapy.

**Additional file 2. **Summary of themes and sub-themes.

## Data Availability

The datasets generated and/or analyzed during the current study are not publicly available due to participant privacy but are available from the corresponding author on reasonable request.
