# Supplementary material for: Challenges and recommendations to improve implementation of phototherapy among neonates in Malawian hospitals
Source: BMC Pediatr. 2022 Jun 27;22:367. doi: 10.1186/s12887-022-03430-y (PMC9235141; doi:10.1186/s12887-022-03430-y)
Supplement: Supplementary file 1 — Additional file 1. Caregiver experiences with phototherapy. [file 12887_2022_3430_MOESM1_ESM.pdf]

Keep probing **why**
** "Can you provide an example?"**

Caregiver experiences with phototherapy

Topic Guide

- *Introduce yourself and ask the participant how they are doing today, etc.*
- *Introduce the project, explain that we are seeking to understand experiences about phototherapy and infant warming and go through the consent form with the participant*
- *Start recording with:* ***date of interview*** *-* ***interview code - name of hospital - gender of participant***

Introduction

1. How are you related to the newborn?
2. Please tell us a little about yourself (what is your/your husband's profession, how many children do you have?)
3. Where are you from and how did you come to the hospital today (what means of transport, how long did it take, cost/availability of transport, were you referred here by another facility?
4. Who is looking after your other children at home? Any concerns about time away from home and/or job?

Phototherapy

5. Please describe how medical staff started phototherapy with you? Did they counsel and ask permission before starting your baby on phototherapy? How did that make you feel?
6. How did medical staff explain phototherapy to you? Why did they say that phototherapy is needed? Do you think that was enough information?
7. Were there any delays in starting your baby on phototherapy? If so, what do you think caused the delay?
8. Do you feel that the staff were sufficiently trained to start and monitor the baby on phototherapy? Why or why not?
9. How often did medical staff check up on (monitor) your baby on phototherapy? Do you think that was enough?
10. What roles and responsibilities do you have as a caregiver in monitoring /looking after your baby while on phototherapy? How do the medical staff support you?
11. How comfortable do you feel about the use of phototherapy for your baby? Please rate how you feel from one (very uncomfortable) to five (very comfortable) and share why.
12. Did you know about phototherapy before your stay here at the hospital? Where did you hear about phototherapy from? What do people say about it?
13. Are there any cultural beliefs about phototherapy and the blue light?
14. What do you think are the benefits of phototherapy? Are there any harms?
15. What challenges do you face while your baby is under phototherapy?
16. What support can medical staff / other mothers in the unit / your family provide for phototherapy?

Closing

17. Thank you. These are all the questions I had for you. Is there anything you would like us to know about your experience with phototherapy?

Healthcare workers experiences with phototherapy

Topic Guide

- *Introduce yourself and ask the participant how they are doing today, etc.*

Keep probing **why**
** "Can you provide an example?"**

- *Introduce the project, explain that we are seeking to understand experiences about phototherapy and infant warming and go through the consent form with the participant*
- *Start recording with:* ***date of interview*** *-* ***interview code - name of hospital - gender of participant***

Introduction

1. What is your current job position and how long have you been working in this unit?
2. How long have you been a health care worker?

Phototherapy

3. How often do you use phototherapy for neonates in this unit?
4. Were you formally trained to use phototherapy for neonates? If so, please describe the training. If not, were you oriented to its use?
5. Do you feel comfortable using phototherapy? Please rate how you feel from one (very uncomfortable) to five (very comfortable) and share why.
6. How do identify neonates that require phototherapy in this unit? Are there guidelines or protocols available? Are these guidelines routinely used?
7. What is the process of starting a neonate on phototherapy? Who decides and who actually puts the neonate on phototherapy?
8. Are there any disagreements between medical staff about starting a neonate on phototherapy? Who owns the decision when something goes wrong?
9. What can cause delays to starting a neonate on phototherapy in this unit? Has there been times when a baby was eligible for phototherapy but was not initiated? If so, why?
10. How do you manage neonatal jaundice when the phototherapy device is not available/functional?
11. When there is a neonate on phototherapy, who monitors and what do they do for monitoring?
12. What can cause delays to monitoring a neonate on phototherapy?
13. What is the process of taking a neonate off phototherapy?
14. How has phototherapy affected the care in your institution? Have you seen any benefits? Any harms?
15. What to caregivers think of phototherapy? What emotions do they feel?
16. Are there any cultural beliefs about phototherapy or the blue light?
17. How do you explain phototherapy to caregivers? What do you think helps caregivers understand phototherapy better? What makes it difficult to explain phototherapy to caregivers?
18. Any examples of caregivers who refused? If so, why?
19. What supplies and equipment are needed to effectively use phototherapy in this unit? In your opinion, are these supplies reliably available? How frequently is the phototherapy device not functional and what happens when it is in need for repair?

Closing

20. Overall, what do you think are the most important challenges to using phototherapy in your unit?
21. What do you think would help staff use phototherapy better in your unit?
22. Do you feel that staff receive the support to they need to effectively use these devices from management?
23. Is there anything you feel has been missing in your training about phototherapy that you wish was part of your training?
24. Thank you. These are all the questions I had for you. Is there anything you would like us to know about your experience with phototherapy?
