# Supplementary material for: Challenges and recommendations to improve implementation of phototherapy among neonates in Malawian hospitals
Source: BMC Pediatr. 2022 Jun 27;22:367. doi: 10.1186/s12887-022-03430-y (PMC9235141; doi:10.1186/s12887-022-03430-y)
Supplement: Supplementary file 2 — Additional file 2. Summary of themes and sub-themes. [file 12887_2022_3430_MOESM2_ESM.pdf]

Summary of themes and sub-themes

Theme	Subtheme	Illustrative quotes	
		**Tertiary hospital**	**District hospital**
Challenge of diagnosing jaundice	Diagnosing on clinical presentation	"It's a bit tricky because we don't have, transcutaneous bilirubinometer that helps us to identify whether the child needs phototherapy or not. Uhh currently, we are using our eyes…We check the baby skin color by pressing on either the forehead or the chest and see whether the urine discoloration is going to come out or not, so that's the thing we are using."(QE-CN-01)	"There is a chart in the nursery that is used because we don't have a transcutaneous bilirubin machine here that helps us to know the levels. So we just use clinical signs, so we know that at this level, it now time to put this one on phototherapy." (cw-co-02)
	Training gaps	"I have seen there is a coding guideline in the nursery where they have documented the values if you are using whether a bilibrinometer or bilirubin levels from the lab and also… where you can check. [For example,] maybe if you can see that this part is looking more jaundiced… If you ask most of the people… they don't really understand most of it… I don't think they (the guidelines) are really used." (QE-RE-01)	"I cannot say I am very comfortable…I need to undergo the formal training… since whenever you are told something by someone, it is always brief… so that you should just have an idea unlike a when it is formal, you know that it goes like this." (CW-CO-01)<br><br>"Not really knowing what jaundice look like … because we were not trained. So everyone rates [the] baby on their own, so the challenge is that to some, it is very difficult to identify the jaundice… Of [the] nurses in our ward, it's only one person who have trained on phototherapy and the other four we did not go to training, so it's a big challenge" (CW-NU-02)
	Disagreements and medical hierarchy	"Maybe a nurse has seen a child maybe while giving medicine that this child is looking like jaundice [and recommends] we should start him on phototherapy, then you find the doctor has seen him and says no he is fine, he should be let out, then you find that maybe the child is out	"A baby came with presentation of jaundice when I was alone on night duty so I initiated the baby on phototherapy and then the clinician came and said, "What were the presentations?" and I explained the indicators I used. I briefed him that

		then you find that after two days he is back with the same problem, so it can happen…The doctor has more power than the nurse, but most of the time, it's that the nurse is working more closely with the patients than the doctors. The doctors can see the child today then he will see him again when, maybe tomorrow his friend will see him, but as a nurse she will see him today, see him tomorrow so you can differentiate that this child at first he looked pink, here we see that he has changed… If something were to go wrong, it would fall on the nurse since she is the one who is small, the one who is below. But if … they measure the bilirubin… so the results are what determine how we manage the child (QE-NU-01)	the baby will benefit from phototherapy but at the end, he discharged the mother." (CW-NU-01)
Challenges for counselling to alleviate caregiver fears about phototherapy	Fears about phototherapy among caregivers	"They were just concerned that isn't this dangerous? Since people, when they hear that a child has gone to phototherapy, they are afraid, so it just scared them" (QE-FG-03)<br><br>"Others say that if a child is on these lights he will never give birth, his what is destroyed, his reproductive organs."(QE-NU-01)<br><br>They become worried as to why their baby is put under the blue light… sometimes they don't expose the baby by covering it." (QE-CN-01)	"Most people … think if we put a baby on that machine it will die. For example, if we tell them of the oxygen machine they say the baby will die. The same thing with this machine (phototherapy); they think the baby will die." (CW-NU-01)<br><br>"Some understand fast [but] some they think that light on phototherapy destroys the baby, so it just depends on guardian (caregiver)…a lot of people saying that when the baby is on phototherapy they don't get well…" (CW-NU-02)<br><br>"I was asking other women, I said, when you are looking at this bulb, how do you look at it? They said, it sucks blood…They say it sucks blood, and the child does not get well." (CW-NU-04)

			"Sometimes when you cover the eyes, it's like the child is being arrested so they wonder why the child can't see them because they would like to see the eyes of their children. They feel like they are being tortured so they are not happy with that. So sometimes if you don't monitor you find that they have taken it off" (cw-co-02)
	Difficult to explain phototherapy in lay language	"They explained that, they put them under electricity that helps to deal with yellowishness" (QE-MG-05)	On what makes it difficult to explain phototherapy to care givers: "I think the vocabulary. They don't really understand how this light works and that becomes difficult. " (cw-dmo-01)
	Family decision-makers and gaps in engaging relatives	"I wouldn't know because as husbands here, we are only given little time just 5 minutes to go and see the baby… it is very limited. I as a father, the experience that I got here is that, as men we are not permitted to have more time there." (QE-MG-03)	"Most of the time, we explain to the mothers…[but] they may ask from other guardians' relatives like their mothers or their in-laws." (CW-NU-01)
Infrastructure and resource gaps	Unreliable electricity at the districts	-	On what can cause initiation delays: "Sometimes electricity…Because here in out unit, when power goes off, it's a problem. We don't have a generator." (CW-CO-01)
	Overcrowding and lack of adequate phototherapy devices especially at the tertiary hospital hospital	"There [was] also another child. They left him (her child) and helped the other one first…This delay was maybe because the other child's condition was worse than mine." (QE-FG-03)<br><br>"There are times you have a lot of babies on phototherapy then all the machines are occupied, then there is another baby who needs phototherapy… Most of the times, they share the phototherapy machines. We can put two babies in two cots and then they are using one phototherapy machine. If the phototherapy	

		machines are so occupied that the baby cannot share with the other baby, we assess the severity of the jaundice as compared to those that have been already started on phototherapy so if somebody's jaundice is resolving we compare with the one that has not started then they switch." (QE-NU-05)	
	Human resource shortages	"I think that they (staff) are trained (to monitor) but …I think that they are too short staffed, what I mean is that there are too many babies for the staff. So the work sometimes becomes overwhelming and sometime you need assistance, some would assist you but some will be angry when you are talking to them, so you just leave them to do their work, but sometimes you need more of their assistance…" (QE-MG-03)	"Not having enough human resource. We don't have a clinician based in nursery full time. We don't have enough nurses, of course there are there that are allocated in the nursery but they have to look for all the babies in the nursery. Sometime they might delay monitoring the baby because the thing they are doing is an emergency or it's a priority." (cw-dmo-01)
Recommendations to improve implementation of phototherapy	Peer support for caregivers with concerns about phototherapy	"Mine was also like this but now he has changed, so that means that your will also change and he will be fine," those are the things that I have heard from my fellow women." (QE-FG-03)	"Mine was also like this but now he has changed, so that means that your will also change and he will be fine," those are the things that I have heard from my fellow women." (QE-FG-03)
	In house maintenance teams	"We have technicians who do come… to see how the machines are working… Whenever we have a fault, I think they are called immediately to see whether that can be repaired or not. (QE-CO-02)	"We called maintenance team to come and fix it the same day… Most of the guys live within the hospital. Even at night, we call them." (CW-NU-01)
	Bilirubin testing	"we use the clinical judgment to put the baby on phototherapy, so I think we need to have maybe sometimes some objective measures of the jaundice of the bilirubin in this child so that we actually put with ease on phototherapy, because just looking at the baby clinically is quite subjective, I think that why sometimes we do	"We do receive support from management but we still need more support like on the part of the device which we use to check the extent of the bilirubin levels or what because sometimes you just put the babies, we just leave them there not knowing which level of bilirubin is how far it dropped" (CW-CN-01)

		have maybe some disagreements, say no this one is very jaundiced somebody says is not, so I think if we can have some bilirubin measuring machines that would be very important “ (QE-CO-02)	
	Reliable electricity	-	“Mostly… the issue of power is a problem. The availability of a generator would be helpful, so that this process should be continuous. As a nursery, we will not benefit through phototherapy alone, CPAP will also benefit” (CW-CO-01)
	More phototherapy devices and accessories	“Maybe if we can have also some… eye pads…because I think most of the times we just improvise....So, you make sometimes something which is too big covering almost the whole face instead of just covering the eyes, so if we can have these eye pads it could be very, very, very important.” (QE-CO-02)	“We don’t have enough phototherapy light, that’s a challenge for us and material to cover the eyes. I think the issue of not having enough phototherapy lights is a bigger issue” (cw-dmo-01)
